# Real-Time Control of a Video Game Using Eye Movements and Two Temporal EEG Sensors

**DOI:** 10.1155/2015/653639

**Published:** 2015-11-15

**Authors:** Abdelkader Nasreddine Belkacem, Supat Saetia, Kalanyu Zintus-art, Duk Shin, Hiroyuki Kambara, Natsue Yoshimura, Nasreddine Berrached, Yasuharu Koike

**Affiliations:** ^1^Department of Neurosurgery, Osaka University Medical School, Osaka 565-0871, Japan; ^2^Intelligent Systems Research Laboratory, University of Sciences and Technology of Oran, 00031 Oran, Algeria; ^3^Department of Information Processing, Tokyo Institute of Technology, Yokohama 226-8503, Japan; ^4^Precision and Intelligence Laboratory, Tokyo Institute of Technology, Yokohama 226-8503, Japan; ^5^Solution Science Research Laboratory, Tokyo Institute of Technology, Yokohama 226-8503, Japan

## Abstract

EEG-controlled gaming applications range widely from strictly medical to completely nonmedical applications. Games can provide not only entertainment but also strong motivation for practicing, thereby achieving better control with rehabilitation system. In this paper we present real-time control of video game with eye movements for asynchronous and noninvasive communication system using two temporal EEG sensors. We used wavelets to detect the instance of eye movement and time-series characteristics to distinguish between six classes of eye movement. A control interface was developed to test the proposed algorithm in real-time experiments with opened and closed eyes. Using visual feedback, a mean classification accuracy of 77.3% was obtained for control with six commands. And a mean classification accuracy of 80.2% was obtained using auditory feedback for control with five commands. The algorithm was then applied for controlling direction and speed of character movement in two-dimensional video game. Results showed that the proposed algorithm had an efficient response speed and timing with a bit rate of 30 bits/min, demonstrating its efficacy and robustness in real-time control.

## 1. Introduction

Electroencephalogram (EEG) is a noninvasive technique for measuring electrical potentials from electrodes placed on the scalp produced by brain activity and some other artifacts such as Electrooculogram (EOG) and Electromyogram (EMG). Nowadays, EEG technique has been used to establish portable synchronous and asynchronous brain-computer interfaces (BCIs). Noninvasive EEG-based BCIs are the most promising interface for space applications. They can be classified as “evoked” or “spontaneous.” An evoked BCI exploits a strong characteristic of the EEG, the so-called evoked potential, which reflects the immediate automatic responses of the brain to some external stimuli. Spontaneous BCIs are based on the analysis of EEG phenomena associated with various aspects of brain function related to mental tasks carried out by the subject at his/her own will.

BCIs offer people with movement disabilities a means of interaction with their environment by translating brain activity into device control [1]. Recently, several BCIs have been developed based on evoked potentials such as P300 and steady-state visual evoked potential (SSVEP) or based on slow potential shifts and variations of rhythmic activity [2]. Many critical issues are faced on the development of a BCI such as classification accuracy, number of degrees of freedom, and training process (i.e., how users learn to operate the BCI). Some researchers have demonstrated that BCI users can learn to control their brain activity through video games [3, 4]. Therefore, EEG-controlled gaming applications can provide strong motivation for practicing. In this respect, a main issue is how to develop medical and nonmedical games to improve the robustness of BCIs with the goal of making it a more practical and reliable technology.

Eye movements and blink artifacts are pervasive problems in EEG-based BCI research [5]. However, the present authors feel that these artifacts are actually a valuable source of information and are useful for communication and control. In this paper, several participants were tested in different real-time experiments on different days to examine the variability and nonstationary nature of EEG signals. This study has shown that the same control performances can be obtained via either EOG or EEG signals with using suitable positions and minimum number of sensors for EEG technique. The control performances of participants were tested in natural environment where they were asked to perform the movements of their eyes, body, and head as naturally as possible.

In Section 2, BCI-based medical and nonmedical games, popular techniques for eye tracking, and hybrid BCIs based on brain activity and eye movement are reviewed. In Section 3, materials and methods for developing several paradigms of real-time experiment are introduced. These experiments are based on real-time classification and control with opened and closed eyes using our proposed algorithm with minimum number of EEG sensors. In Section 4, results of eye movements' classification and video game control are presented. Advantages and disadvantages of the proposed idea in different scenarios are discussed with detailed aspects in Section 5. Finally, conclusion and prospects of future work are given in Section 6.

## 2. Related Work

### 2.1. BCI-Based Games

Simple BCI-based games can help inexperienced users control via brain activity. Games based on EEG have been designed to increase the intensity or duration of attention, increase the speed and accuracy of brain-signal control, and improve other capabilities [2]. Two types of BCI-based games are frequently seen: medical and nonmedical games. For medical purposes, Lalor et al. [3] describe a game intended to improve the concentration needed to operate a BCI that uses SSVEP. Other medical games were designed to encourage rapid generation of motor imagery-based BCI commands and enhance the user's experience [4]. For entertainment purposes, several BCI-based games were developed, for example, the one based on popular video game “Tetris”, playing pinball, and dancing robot [6–8]. Most of these nonmedical games are based on concentration level of the player [8].

### 2.2. Eye Tracking

In daily life conversation, eye movements play an important role in interaction with environment by indicating a person's direction and level of attention. Fortunately, most of handicapped people can still control their eyes. Thus, eye movement can be an additional option to improve their quality of life. Eye movements can be measured as EOG signals or via cameras and applied to communication or control systems [9–19]. Both methods have their respective merits and demerits (Table 1).

### 2.3. Hybrid BCIs

Recent studies have shown that EOG signals acquired using EEG technique with a minimal number of EEG sensors around the frontal lobe or ears are practical to detect and classify eye movements [20–23]. Therefore, brain activity was not extracted from EEG to be used as additional information. Hybrid BCIs offer a potentially effective control for complex systems through the combination of brain- and non-brain-based activities. Wheelchair control, for example, requires multiple degrees of freedom and fast intention detection, making solely EEG-based wheelchair control a challenge. Each type of BCIs has its limitations, but a hybrid BCI combines different approaches to utilize the advantages of multiple modalities [24, 25].

A hybrid BCI combining motor imagery and P300 was proposed in Li et al. [26]. It was further used to control the direction and speed of a wheelchair in Long et al. [27]. However, a fast and accurate design for the stop command and the forward and backward commands has not been obtained. Wang et al. [28] proposed asynchronous wheelchair control with a hybrid EEG-EOG BCI, combining motor imagery, P300 potentials, and eye blinking. Their experimental results not only demonstrated the efficiency and robustness of brain-controlled wheelchair systems but also indicated that the participants could control the wheelchair spontaneously and efficiently without any other assistance. However, Wang et al. used only a single eye movement, eye-blinks. Here, we show that more than six classes of eye movements can be classified and used for real-time control, demonstrating the utility of EOG signals in EEG data. Hereafter we provide explanations of the experiment paradigm, EEG recording, and real-time video game control, describe and discuss our classification and control results, and then conclude with future prospects.

## 3. Materials and Methods

### 3.1. Experimental Paradigm

Five participants (4 males, 1 female) with a mean age of 26.2 years (standard deviation (SD): 2.5) were seated in a chair and instructed to watch a monitor screen located in different positions away at eye level. All subjects reported normal or corrected-to-normal vision and had no prior BCI experience. One of them suffers from Amblyopia (vision problem also called lazy eye). Subjects participated in a real-time test experiment, followed by an eye-controlled video game. The real-time test experiment was created to evaluate the performance of the proposed algorithm. Participants then played the video game using eye movements. In this study, classification accuracy was calculated using the real-time test experiment, and control performance was evaluated using the video game.

**Figure 1:**
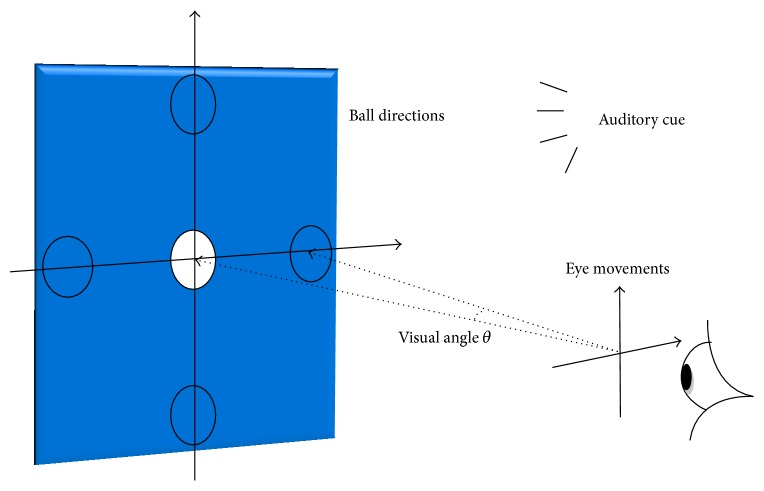
Real-time experiment for controlling a white ball with opened and closed eyes based on eye movement.

**Figure 2:**
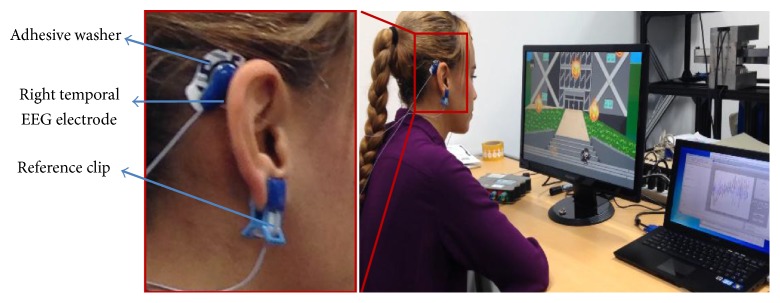
Setup for EEG recording and game control.

In the real-time test experiment (Figure 1), five participants were asked to move a white ball to five positions (up, down, left, right, and center) using eye movements or change its color by blinking. The participants did not move their eyes on consistent time interval during control period. Subjects performed ten runs (10 trials for each eye movement), with each run lasting 60 s. During the first 10 s, the participants were asked to fixate a white ball in the center. Then they were asked to move the white ball to one of the four cardinal directions (up, down, right, or left) using eye movements. In the last 10 s, the participants were asked to blink three times to change the color of the ball from white to yellow. The participants were asked then to move a white ball to five positions with closed eyes using voice instructions to show the feasibility of sending commands in real time by blind persons for autonomous eye movement based control systems. After testing the performances of the proposed algorithm in this real-time experiment, the participants were able to play a video game during 20 minutes using eye movements without any training or calibration phase. In real-time control of video game, the subjects can move their head position and direction and watched the motion of both a game character and meteors in various timings.  Figure 2 shows the experiment framework and electrode positions for eye-controlled gaming.

### 3.2. EEG Recording

EEG signals were acquired during real-time experiments using a g.USBamp system (g.tec medical engineering, Austria) at a sampling frequency of 256 Hz. Two EEG electrodes were applied on the upper area behind the left and right ears (Figure 2). This proposed position was favorable because it was not obstructed by hair and allowed for capture of EOG without the discomfort that might occur with electrodes on the face. A clip electrode was attached to the right earlobe as reference and ground electrode was placed on the forehead. To prevent muscle artifacts, participants were asked to avoid strong blinking and head movements.

An 8th-order Butterworth band-pass filter with a lower cut-off frequency of 0.5 Hz and an upper cut-off frequency of 100 Hz was applied to the recorded signals. A 4th-order 48–52 Hz notch filter was used to suppress 50 Hz power-line noise.

### 3.3. Classification Algorithm

**Figure 3:**
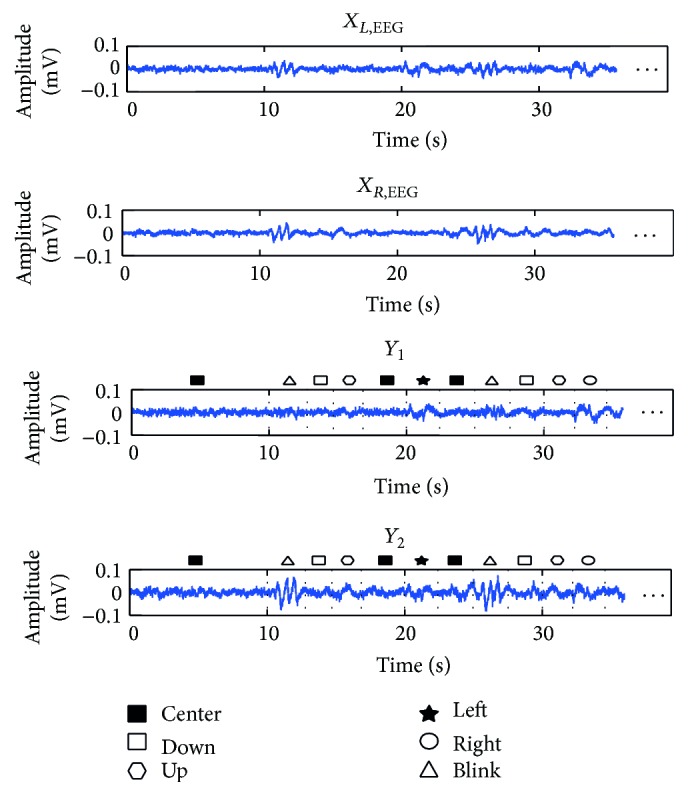
Example of raw EEG signals *X*_*L*,EEG_ and *X*_*R*,EEG_, from the left and right electrodes, respectively, and processed signals *Y*_1_ and *Y*_2_. The symbols represent blink and eye movement classes.

After recording the EEG signals from right and left hemispheres, a real-time algorithm was applied to distinguish between six classes: blink, center, right, left, up, and down. Signal data were sent from the amplifier to the computer in 1 s blocks. The EEG signals were separated into low and high frequency components to separate EOG activity from brain activity (1). Thus, 4th-order Butterworth high and low pass filters with cut-off of 10 Hz were used to decompose EEG signals into two frequency bands: low [0.5–10 Hz] and high [10–100 Hz]. For preprocessing phase, the baseline artifact was corrected by subtracting the smoothed signal with its mean (2). In the current study, EOG signal and eye-blink artifact included in observed EEG signal were used as valuable sources of information:
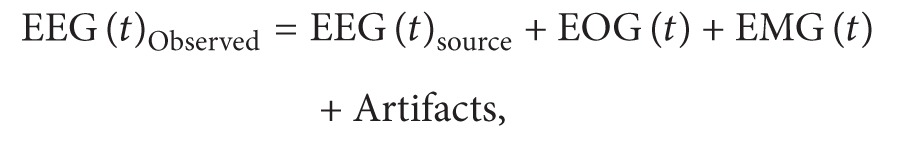
(1)

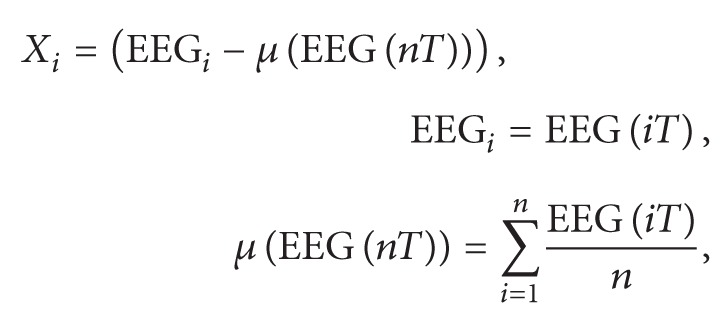
(2)where 1/*T* is sampling frequency of the EEG signal (*t* = *nT*, *n* = 1,2,…, 256). After baseline correction, two signals *Y*_1_ and *Y*_2_ were calculated (3). *Y*_1_ maximized the margin between classes left and right by using the difference between the left (*X*_*L*,EEG_) and right (*X*_*R*,EEG_) electrode signals. *Y*_2_ distinguished between classes up and down using the smoothed sum of the two electrodes. A real-time detection was added before classification phase because the length of time interval of eye movements was not fixed. The length of time interval was varying depending on each trial of natural eye movement and control timing for each participant (Figure 3):

(3) $$\begin{array}{c} Y_{1}=X_{L,\text{EEG}}-X_{R,\text{EEG}},\\ Y_{2}=X_{L,\text{EEG}}+X_{R,\text{EEG}}. \end{array}$$

In our previous work [20], we tested an offline algorithm for classifying between eye movements in four cardinal directions using area under the curve for signals *Y*_1_ and *Y*_2_. Then we added features for online discrimination between six classes of eye movements [21]. Signals corresponding to each eye orientation have a specific shape (Figure 3), and the blink pulse is similar to a Gaussian pulse. The wavelet scalogram was used for detection phase of eye movement. We applied a continuous wavelet transform on signals *Y*_1_ and *Y*_2_. For *a* scale parameter, *a* > 0, and position, *b*, the CWT is

(4) $$\begin{array}{c} {\text{EEG}}_{a,b}\left(\omega \right)=C_{a,b}=\overset{1\;s}{\underset{0}{\int }}\text{EEG}\left(t\right)\psi_{a,b}\left(t\right)dt,\\ \psi_{a,b}\left(t\right)=\frac{1}{\sqrt{\left|a\right|}}\psi^{*}\left(\frac{t-b}{a}\right), \end{array}$$

where *C*_*a*,*b*_ is the continuous wavelet transform coefficients, *a* is positive and defines the scale, *b* is any real number and defines the shift, *ψ*_*a*,*b*_(*t*) is a wavelet function of Haar, *∗* denotes the complex conjugate, and EEG(*t*) is *Y*_1_ or *Y*_2_ signal. CWT is a real-valued function of scale and position because the signal EEG(*t*) is real-valued. By continuously varying the values of the scale parameter, *a*, and the position parameter, *b*, we obtained the wavelet coefficients. Then the wavelet scalogram was obtained by computing:

(5) $$\begin{array}{c} S=\left|\text{coefs}*\text{coefs}\right|,\\ E=\sum S\left(:\right), \end{array}$$

where coefs is the CWT coefficients, *S* is the energy for each wavelet coefficient, and *E* is the energy of the wavelet coefficients for 1 s. For both *Y*_1_ and *Y*_2_ signals, area under the curve of the positive and negative peaks was used for feature extraction. The area was calculated over a 200-ms window centered on the position of maximum wavelet coefficient using the trapezoidal method. The waves were situated in between the maximum wavelet coefficient.

Hierarchical classification was used to discriminate between patterns obtained from *Y*_1_ and *Y*_2_ signals. Fixed thresholds for four features, maximum wavelet coefficient, area under the curve, amplitude, and velocity, were set prior to the real-time experiments by calculating their means and standard deviations based on our previous studies [20, 21]. The classification results were converted into vectors to produce binary outputs. These outputs were used to move a cursor on a screen and control a video game.

### 3.4. Eye-Controlled Game

**Figure 4:**
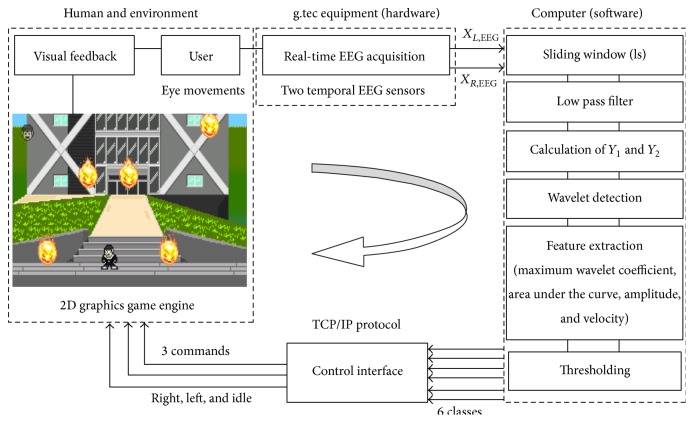
Control flowchart for the real-time eye-controlled game.

To demonstrate the efficacy of eye-based control in real world applications, we created a simple game. The game was an obstacle evasion 2D platformer game. In the game, a character had to run in two directions to avoid being hit by falling meteors which appeared in semirandom sequences on a closed stage. The character's movement was controlled with the player's eye movements (Figure 4).

The implementation of the game was divided into two modules, the EEG signal classification module and the graphic game module. The EEG signal classification module was implemented in MATLAB (MathWorks, Natick, MA), the graphic game module was implemented using the Unity 4 game development ecosystem (Unity Technologies, San Francisco, CA), and the game's logic was written in C#. The two modules interfaced with each other via TCP/IP protocol.

### 3.5. Character's Movement Mechanism

The classification module was capable of discriminating between 6 eye movement classes (left, right, center, up, down, and blink). However, the video game required only 3 commands: “left,” “right,” and “idle,” so the left and right classes were mapped to their respective commands and the remaining classes were mapped to the “idle” command. The character's direction and speed were defined by the sign and magnitude of a unit vector, respectively. Initially, the vector was set to 0, so the character stood still when the game started. A positive vector made the character move to the right, and a negative vector made it move to the left. Left commands from the classification module decreased the vector magnitude by 0.1 units, while right commands increase the magnitude by 0.1 units. The character's speed changed by increasing or decreasing a fixed magnitude for character's acceleration. Commands were received discretely every 1 s to control the character's motion continuously. So the character's movement direction was determined as the dominant command in a given command sequence window. This approach was used to compensate the natural sudden change in eye movement direction when the eyes move back to the center position. The idle command was different from the movement commands in that the vector magnitude was set to 0 immediately after the idle command was received, allowing the character to stop when the player intended with no delay. The maximum speed of the meteors was defined by a vector of units 0.1, and the initial speed was 0. Acceleration downwards was defined as a vector of units 0.01. The number of meteors was semirandomized, but no more than 5 appeared at one time. There were 5 meteors release points lined up evenly across the top of the screen (Figure 4). Each point had its own set of semirandomized release times and delay values. We set the values to release, on average, 3 meteors per repetition. Difficulty was controlled by the number of meteors and their speed. Therefore, classification accuracy was based on stopping and moving the character and not on avoidance of the meteors.  Table 2 summarizes the actions to be performed by the character shown in Figure 3 corresponding to each eye movement.

### 3.6. Evaluation Criteria

The performance of the proposed algorithm was evaluated in the real-time experiments by calculating the classification accuracy for six and three classes based on success or failure to move the ball or character to a desired direction, respectively. For control of the video game, precision, sensitivity, and specificity were calculated for three classes (right, left, and idle) such that

(6) $$\begin{array}{c} \text{Precision}=\frac{\text{TP}}{\text{TP}+\text{FP}}\times 100,\\ \text{Sensitivity (Recall)}=\frac{\text{TP}}{\text{TP}+\text{FN}}\times 100,\\ \text{Specificity}=\frac{\text{TN}}{\text{TN}+\text{FP}}\times 100, \end{array}$$

where TP is the number of true positives, TN is the number of true negatives, FP is the number of false positives, and FN is the number of false negatives.

## 4. Results

Calculating *Y*_1_ and *Y*_2_ resulted in unique signatures for each class (Figure 3) which could be exploited for classification.  Table 3 shows a confusion matrix of the six classes and accuracies averaged across five participants (16.67% chance level). All participants demonstrated reliable control of the white ball in the first experiment, achieving an average accuracy of 77.33 (SD: 2.52%). The proposed algorithm showed high classification accuracy for right, left, center, and blink classes using 0% of the data for training or calibration phase and 100% of the data for testing.

We used a one-way ANOVA to evaluate real-time classification results of the six eye movement classes among participants. No significant differences were observed between participants for accuracy (*F*(4,25) = 0.06, *P* = 0.993).

Table 4 shows a confusion matrix of the five classes (up, down, left, right, and center) and accuracies averaged across five participants with closed eyes using the same threshold values. All participants resulted in almost the same classification accuracy with opened or closed eyes, achieving an average accuracy of 80.2% (SD: 1.87%) using auditory feedback with closed eyes (20% chance level) with no significant difference between participants (*F*(4,20) = 0.08, *P* = 0.989).

We observed that classification accuracies using closed eyes of “center” and “up” classes were increased compared to the case of using opened eyes. This accuracy difference between up class in the case of “opened eyes” and “closed eyes” is due to similarity of the wave shape of “up direction” and “eye-blink” signals. The “up” and “blink” classes were combined in one class in eye-closed experiment. Therefore, the classification accuracy of up class was increased. The accuracy of center class was also increased because there is no noise related to the visual information. The algorithm showed a high accuracy and robustness using a single trial for controlling the white ball in real time in the opened- and closed-eyes situations with no significant difference between all users using the same thresholds values for all of them. The participant with Amblyopia disease showed the lowest classification accuracy compared to others due to the difficulty in moving his eyes correctly.

In real-time control of video game, the subjects can move their eyes position and direction and watched the motion of game character and meteors in the various timings.  Table 5 shows precision, sensitivity, and specificity for each participant. These values were calculated based on accuracies (Table 2), with up, down, center, and blink classes considered as the idle class. Average sensitivity was over 90%, and participant 3 achieved an accuracy of around 100% using a single trial to make decision. No significant differences were found between participants for accuracy (*F*(4,10) = 1.23, *P* = 0.359), sensitivity (*F*(4,10) = 0.63, *P* = 0.653), or specificity (*F*(4,10) = 0.94, *P* = 0.478).

Response speed and timing are also important in full control of a BCI. Using serial communication, the classification algorithm processed 60 bits/min, but the control algorithm processed the first bit and ignored the second. Therefore, the bit rate for controlling the video game was 30 bits/min. The proposed algorithm was useful in classification accuracy and time-saving because the main problem faced by real-time application is the computing and processing time.

## 5. Discussion

In this study, subjects were able to perform real-time control of an interface using six eye movements and play a video game with three eye movement based commands. Because the resting position of human eyes is forward-facing, we return our eyes back to the center position after looking at any other direction. This action would have resulted in classifications opposite to the intended direction and, in turn, adversely affect interface control. To solve this problem, the game character's movements did not follow the commands sent from classification module verbatim. The movement of the character was defined as a unit vector of acceleration along *x*-axis, with “right” being positive values and “left” being negative values. Movement commands gradually increased the acceleration value in the intended direction. This technique reduced the effect of the eyes returning to the center position. For the “idle” or stop command, which required an immediate response, the movement vector magnitude was immediately returned to zero to stop the character stop as soon as the player intended.

For classes up and down, even though the two sensors were located at the same points behind the right and left ears, we were able to obtain discriminable EOG activity. We believe that the eyes did not move at mirrored angles across the central axis. This dissimilarity likely made detection of up and down directions possible and was amplified by calculating *Y*_2_.  Table 6 summarizes the advantages and disadvantages of the proposed robust real-time control system based on EEG signal.

Since the magnitude of the electrical signal generated by the eye movement depends on the angular velocity, many researchers have used a big visual angle of between 30° and 45° to get a high accuracy for detecting the directions or positions of the eye movement [12, 29–33]. This large visual angle is not suitable for daily life applications because it leads almost immediately to eye fatigue, exhausting the user. Comparing the real-time results using opened eyes from this study with those of our previous offline and online classification work [20, 21], we found that classification accuracy using a small visual angle decreased from almost 90% to 77%. This was likely due to the complexity of the real-time application, environment conditions, and users' behavior. The participants were asked to make themselves comfortable and perform the movements as naturally as possible. There were instances where eye movements were misclassified, but the signal data showed no serious influence by head or body movements. Although future versions of the proposed algorithm would benefit from an automatic thresholding subroutine instead of a calibration phase, results showed that the current algorithm holds promise in real-time applications.

Through this work, we can help not only handicapped people but also the blind persons to use their eye movements using voice commands with auditory feedback for controlling smart-home applications. For able-bodied users, the idea of sending commands with closed eyes can decrease the fatigue issue related to rich detailed visual environments. In some special eye movement based applications, the visual information can be replaced by information from the tactile, olfactory, or auditory senses such as the case of reducing or increasing the room temperature and the volume of music. We sought to contribute to the development of noninvasive, asynchronous, and hybrid BCIs combining brain activity and eye movements. This kind of BCI could offer utility in daily life applications and practical machine control. Though most approaches in the BCI field focused solely on brain activity, we see an opportunity for advancement of this field by combining EEG and EOG. This approach could be used to assist both able-bodied and disabled persons with high efficiency compared to existing BCIs.

## 6. Conclusions 

This paper presented asynchronous and robust real-time control of a video game through eye movements detected using two temporal EEG sensors. The algorithm was designed for multiclass control in a visually elaborate immersive 2D game. Results of the study indicated that successful multiclass control is possible using suitable position of sensors to detect and classify eye movements in opened- and closed-eyes situations.

In the near future, for rehabilitation, medical therapy, and entertainment, we would like to design portable, noninvasive, and asynchronous hybrid EEG-EOG-based games and smart-home applications using minimum number of wearable wireless sensors.

## Supplementary Material

Control video game with eyes using two temporal EEG sensors (A real-time BCI).

## Figures and Tables

**Table 1:** Comparison table of EOG- and video-based eye-tracking techniques.

Criteria	EOG electrodes	Video-based eye tracking
Intrusiveness	Intrusive with electrodes attached to the face (i.e., electrodes mounted on the skin around the eye).	Intrusive for cameras attached to glasses; nonintrusive for cameras mounted independently.

Complexity	(i) Electrodes number reduces the portability of the technique (many electrodes attached on the face). (ii) EOG is a simple and easy method to measure eye movements and it is still commonly used clinically for testing eye movements in patients.	(i) The algorithm complexity of the image processing system. (ii) Calibration is a crucial problem: head distance, head and pupil range of rotation with respect to sagittal plane of the body must be estimated (in some case manual corrections are still needed).

Influence of noise	Facial muscles (EMG signal) can be influenced on EOG signal.	(i) Light: big problem for image processing. (ii) Head movement: must keep your eyes open and in the vision field of the camera. (iii) Hard to use it in real-life application (outside environment).

Processing time	Fast: training or calibration phase needed.	Long: training or calibration phase needed; image processing takes much memory.

Classification accuracy	High, but related to visual angle, number of electrodes, and algorithm applied.	High, but related to head angle, user environment, and algorithm applied.

**Table 2:** Vocabulary of real-time commands for eye-controlled gaming.

	EEG signal	Character action
Command 1	Eyes moving to the up position and then returning back	Stop
Command 2	Eyes moving to the down position and then returning back	Stop
Command 3	Eyes moving to the left position and then returning back	Move at the left side
Command 4	Eyes moving to the right position and then returning back	Move at the right side
Command 5	Blinking	Stop
Command 6	No eye movement (fixation)	Stop
Command 7	Two successive similar movements of eyes to the left or right direction	Increase the speed
Command 8	Two successive opposite movements of eyes such as moving to the left then right position or vice versa	Decrease the speed

**Table 3:** Confusion matrix of the six classes and accuracies (rounded %) averaged across all participants.

	Up	Down	Right	Left	Center	Blink
Up	**42**	14	0	2	28	14
Down	6	**50**	0	0	24	20
Right	0	0	**96**	4	0	0
Left	0	0	0	**100**	0	0
Center	4	0	0	2	**88**	6
Blink	0	0	6	4	2	**88**

**Table 4:** Confusion matrix of the five classes and accuracies (rounded %) averaged across all participants with closed eyes.

	Up	Down	Right	Left	Center
Up	**65**	5	0	10	20
Down	12	**46**	19	15	8
Right	0	0	**98**	2	0
Left	0	0	2	**97**	1
Center	4	1	0	0	**95**

**Table 5:** Precision, sensitivity, and specificity values (rounded %) for each participant during real-time game play.

	Right	Idle	Left
Participant 1 (M)	100/90/100	95/100/100	70/100/94
Participant 2 (M)	83.3/100/95.9	92.5/97.4/100	90.9/100/97.9
Participant 3 (M)	100/100/100	100/100/100	100/100/100
Participant 4 (F)	90.9/100/98	95/100/95.3	90.9/100/98
Participant 5 (M)	100/90.9/100	100/100/100	90.9/100/97.5

**Table 6:** Advantages and disadvantages of eye movements classification based on EEG signal.

Criteria	Advantage	Limitation
Visual angle	A small visual angle between 5° and 10° was used to decrease fatigue issue (a large visual angle of 30° or more is required to detect eye movement in most research using EOG signals. This large visual angle leads almost immediately to eye fatigue, exhausting the user).	It becomes difficult to detect eye movements if the visual angle is less than 5°.

User	Several participants were tested (offline [20], online [21], and in different real-time experiments in this study) on different days to examine the variability and nonstationary nature of EEG signals.	Absence of testing the proposed algorithm on handicapped users.

Sensors position & number	(i) The position of sensors around the ears is more robust to muscles activity noise (body or head movements do not influence so much the classification accuracy). (ii) Two temporal EEG sensors were used (4 attached sensors on the face are used as minimum requirement to get good classification accuracy in EOG technique).	A low-cost wireless device based on the proposed idea is not yet developed.

Comfort and portability	The most suitable sensors position for daily life applications to record eye movements compared with EOG sensors (the sensors can be attached to the end of the glasses arms (temples), headset, and headband).	Less comfort [21].

Real-time classification	(i) Single trial was used for real-time classification. (ii) No training or calibration phase was added before real-time classification (fixed and common thresholds for all subjects were used). (iii) No fixed time interval for eye movements (the user is free to move his/her eyes and send commands at any moment). (iv) Six classes were distinguished using a linear clarifier. (v) Eye movements were detected and classified in open- and closed-eyes cases. (vi) The proposed algorithm was tested in several real-time scenarios.	Using average or loop to make a decision or machine learning methods can improve the classification accuracy but decrease the response time [9–13, 29].

Real-time control	(i) Asynchronous control (the user can send commands even with closed eyes using noninvasive technique). (ii) The classification results were used for full control of continuous character's movement in 2D video game. (iii) The bit rate for controlling the video game was 30 bits/min.	For each application, we need to develop an interface between classification results and the controlled device.

Classification accuracy	Classification accuracy with chance level of 16.67% was greater than 70%, the suggested minimum for reliable BCI control with chance level of 50% [34].	As same as EOG technique [20].
